# The influence of HIV disease events/stages on smoking attitudes and behaviors: project STATE (Study of Tobacco Attitudes and Teachable Events)

**DOI:** 10.1186/1471-2458-14-149

**Published:** 2014-02-11

**Authors:** Damon J Vidrine, Faith E Fletcher, Meredith K Buchberg, Yisheng Li, Roberto C Arduino, Ellen R Gritz

**Affiliations:** 1 Department of Behavioral Science, The University of Texas MD Anderson Cancer Center, P.O. Box 301439, Unit 1330, Houston, TX 77030-1439, USA; 2 Division of Community Health Sciences, University of Illinois at Chicago School of Public Health, 1603 West Taylor Street, MC 923, Chicago, IL 60612-4394, USA; 3 Department of Biostatistics, The University of Texas MD Anderson Cancer Center, P.O. Box 301402, Unit 1411, Houston, TX 77230-1402, USA; 4 Division of Infectious Diseases, The University of Texas Health Science Center at Houston Medical School, 6431 Fannin, MSB 6.120, Houston, TX 77030-1439, USA

**Keywords:** Smoking cessation, HIV/AIDS, Cell phones, Underserved populations, Teachable events

## Abstract

**Background:**

Given the increase in life expectancy among HIV-positive individuals attributable to antiretroviral therapies, cigarette smoking now represents one of the most salient health risks confronting the HIV-positive population. Despite this risk, very few efforts to date have been made to target persons living with HIV for smoking cessation treatment, and no efforts have been made to explore the role of cognitions and HIV disease events/stages on smoking outcomes. The purpose of the study, Project STATE (Study of Tobacco Attitudes and Teachable Events), is to prospectively examine the relationship between HIV events/stages, perceived impact of HIV disease, attitudes about cigarette smoking, and smoking behaviors.

**Methods/Design:**

This study employs a prospective design. Patients are recruited at the time of their first physician visit at a large inner city HIV-clinic – Thomas Street Health Center (TSHC). Consenting participants then complete a baseline assessment. All participants are offered standard care smoking cessation treatment. Follow-up assessments are completed on four subsequent occasions: 3, 6, 9, and 12 months post-baseline. These follow-up assessments are scheduled to coincide with routine clinic appointments with their TSHC physicians. In addition, each participant is given a prepaid cell phone at the time of enrollment and asked to complete brief phone assessments weekly for the first three months of the study period.

**Discussion:**

By evaluating events/stages of HIV disease as potential teaching moments for smoking cessation, findings from this study could be used to develop treatments tailored to an individual’s stage of HIV disease. This study design will enable us to carefully track changes in smoking behavior over time, and to link these changes to both the course of HIV disease and/or to the participant’s’ perceived impact of HIV. By identifying optimal time points for intervention, the findings from this study will have the potential to maximize the efficiency and efficacy of cessation treatments delivered in resource-limited settings. In addition, the findings will be instrumental in identifying specific constructs that should be targeted for intervention and will provide a strong foundation for the development of future cessation interventions targeting smokers living with HIV/AIDS.

## Background

Available data suggest that the prevalence of cigarette smoking is significantly higher in HIV-positive populations compared to the general population in the US (currently estimated to be 19.0%) [1]. Data from one of our earlier studies indicated that almost 50% of individuals receiving treatment at a large, Houston, Texas-area HIV/AIDS clinic were current smokers. Similarly high rates of smoking (50-65%) have been reported by others [2,3]. While the exact reasons for the elevated prevalence of smoking in the HIV-positive population are not known, there are several plausible explanations. Individuals of low socioeconomic status are both more likely to smoke and more likely to become infected with HIV. Other variables associated with both smoking status and HIV include gay or bisexual orientation [4,5], alcohol and/or illicit drug use [6], and depression [7].

While smoking is a well-established risk factor for numerous adverse health outcomes (e.g., cancer, stroke, heart disease, and chronic obstructive pulmonary disease), a growing body of literature provides strong evidence that people living with HIV/AIDS (PLWHA) are especially at risk. For example, HIV-positive smokers have an increased incidence of several pulmonary diseases, including cryptococcus [8], invasive pneumococcal disease [9,10], and spontaneous pneumothorax [11]. Even in the absence of an AIDS-related pulmonary condition, HIV-positive smokers are significantly more likely to experience respiratory symptoms compared to HIV-negative smokers [12]. Smokers living with HIV/AIDS also have an increased risk of several HIV/AIDS-related oral diseases [13-15]. In fact, several recent studies indicate that smoking among PLWHA represents one of the leading causes of morbidity and mortality, and effective smoking cessation efforts could significantly increase life expectancy [16,17].

The data supporting the adverse effects of cigarette smoking and elevated prevalence of smoking among PLWHA are striking. Given the increase in life expectancy among HIV-positive individuals attributable to antiretroviral therapies, cigarette smoking now represents one of the most salient health risks confronting the HIV-positive population. Despite this risk, very few efforts to date have been made to target PLWHA for smoking cessation treatment, and no efforts have been made to explore the role of cognitions and HIV disease events/stages on smoking outcomes. Thus, the overarching goal of this National Cancer Institute R01-funded study (5R01 CA132636-04) is to prospectively explore the relationship between HIV events/stages, perceived impact of HIV disease, attitudes about cigarette smoking, and smoking behaviors. The specific aims of the study are to: 1) assess the relationship between HIV disease event/stage (i.e., diagnosis, stable, and progressive disease) and smoking outcomes (i.e., intention to quit, number of quit attempts, and cessation outcomes); 2) evaluate perceived impact of HIV as a potential mediator of the association between disease stage and smoking outcomes; 3) evaluate potential mediators (i.e., attitudes about smoking) and moderators (i.e., perceived control and social norms) of the relationship between perceived impact of HIV-disease and smoking outcomes; and 4) describe, in detail, the smoking behavior of individuals within the first three months after study enrollment.

## Methods/Design

### Project overview

Project STATE (Study of Tobacco Attitudes and Teachable Events) is currently underway and employs a prospective design to examine the relationship between HIV events/stages, perceived impact of HIV disease, attitudes about cigarette smoking, and smoking behaviors. To accomplish this goal, we identify and recruit smokers from Thomas Street Health Center (TSHC), a Harris Health clinic in Houston, TX that provides primary and specialty HIV care to approximately one third of PLWHA in Harris County. TSHC provides care to approximately 5,000 PLWHA. After being offered a brief standard care smoking cessation intervention (see Procedures section), research participants complete a computer-administered assessment designed to measure perceived impact of HIV, attitudes about smoking, and current smoking behavior at the time of study enrollment. Participants are then followed over a one-year period and asked to complete follow-up computer-administered assessments at three-month intervals. In addition to the clinic-administered assessments, participants complete brief, weekly cell phone assessments for the first 3 months following study enrollment. The phone-based data collection approach enables us to gather detailed information on both the magnitude and duration of the teachable moment effect in this critical period of time following HIV care initiation. Finally, research staff conduct detailed electronic medical record reviews to document relevant disease- and treatment-related variables.

### Study site and participants

The University of Texas MD Anderson Cancer Center Institutional Review Board approved the study. Eligible participants are 18 years of age or older, new patients of the TSHC clinic, self-reported current smokers at the time of enrollment, able to perform written informed consent and English or Spanish speaking. Participants are excluded if their physician deems them ineligible due to medical or psychiatric conditions.

The target sample size is 450 participants. This estimate is based on the number of newly diagnosed, antiretroviral naïve patients seen at TSHC each month (n = 50), the expected prevalence of current smoking (50%), and the expected participation rate. Each month, approximately 75 new patients are seen at TSHC. Of these, approximately 50 will meet our operational definition of newly HIV diagnosed (i.e., diagnosed with HIV within the past 3 months and antiretroviral naïve).

### Procedures

#### Recruitment

New clinic patients are identified through electronic medical record review. Research staff approach all new patients at their first physician visit and assess current smoking status (i.e., smoked 100 or more cigarettes in lifetime, and smoke every day or most days). Patients that report being a current smoker and meet the other eligibility criteria are offered enrollment.

#### Baseline assessment

Research staff administer an audio computer assisted self-interview (ACASI) to all participants (see Measures section below for a full description of interview measures). The interview takes approximately 30–45 minutes to complete and answers are recorded directly into a computerized database that contains programmed logic checks and skip patterns. At this time, participants also complete the Mini Mental Status Examination and a breath carbon monoxide test. After completing the entire baseline assessment, participants are compensated with a $25 gift card.

#### Follow-up assessments

Follow-up assessments are conducted at TSHC at 3, 6, 9, and 12 months post-baseline. These assessments are scheduled to coincide with scheduled clinic appointments in order to reduce study-related burden. Procedures to reduce attrition include: 1) reminder phone calls; 2) offering follow-up assessments on different days/times to accommodate different schedules; and 3) obtaining the names and phone numbers of at least three collaterals (i.e., friends or relatives). Follow-ups mirror the baseline assessment, consisting of the ACASI, and carbon monoxide (CO) test. Participants receive a $25 gift card for each completed follow-up assessment.

#### Cell phone assessments

A novel component of our proposed design is cell phone data collection, which occurs weekly during the first 3 months of study enrollment (i.e., the 3-month period of time following the HIV diagnosis). We hypothesize that the time of HIV diagnosis (or initiation of HIV care) is the strongest teachable moment for smoking cessation. Thus, the cell phone assessments allow for the collection of detailed information on both the magnitude and duration of the teachable moment effect in this critical period of time. These brief assessments collect information on the participant’s perceived disease impact, attitudes about smoking (i.e., quit motivation and risk perceptions/outcome expectancies) and smoking outcomes (smoking status and intention to quit). To ensure feasibility of this methodological approach, we provide prepaid cell phones to all participants. Prepaid minutes are loaded onto these phones, as needed, to complete the brief assessments. Following completion of the study, participants are allowed to keep the phones. In previous studies, we have used this approach very effectively.

#### Smoking cessation treatment

The goal of the study is not to assess the efficacy or effectiveness of a smoking cessation intervention. However, due to the overwhelming evidence of adverse effects for continued smoking among PLWHA, all participants are provided with information about the smoking cessation resources available at the clinic. Participants who continue to smoke at the time of follow-up assessments are again offered this usual care treatment.

### Conceptual framework

**Figure 1:**
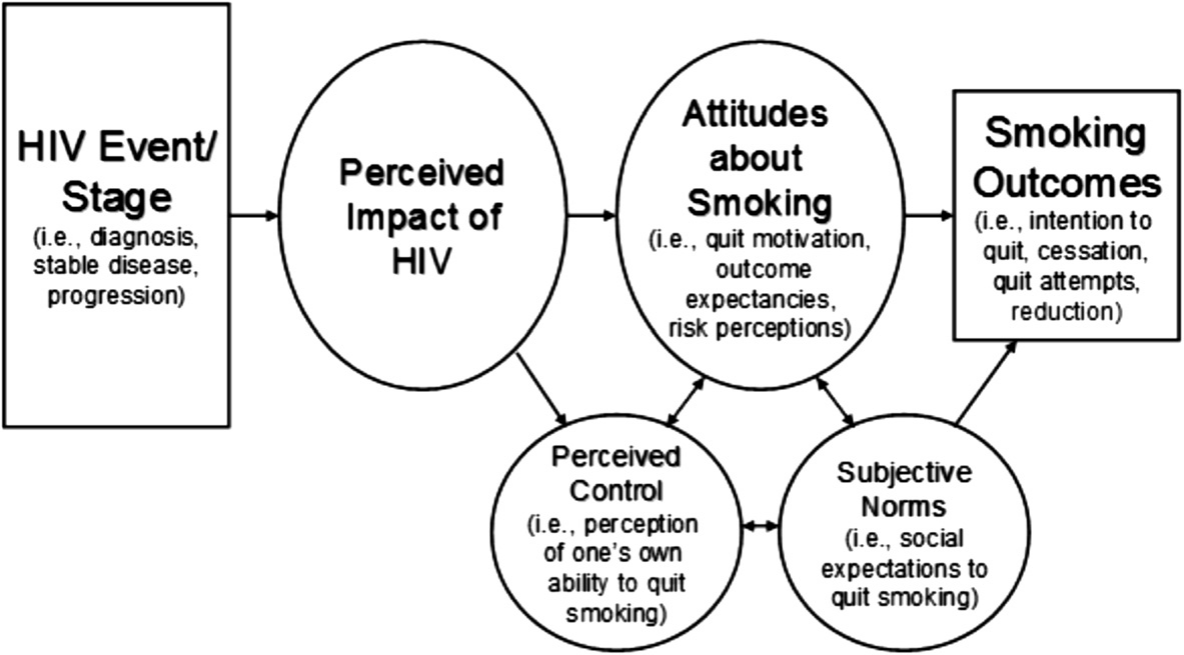
Conceptual framework for the association between HIV events/stages and smoking.

The conceptual framework for this study (see Figure 1) draws heavily from the teachable moment heuristic proposed by McBride and colleagues [18], and from major theories of health behavior change such as the Health Belief Model [19], Social Cognitive Theory [20], the Theory of Reasoned Action/Planned Behavior [21], and stress responses (i.e., the Cognitive Activation Theory of Stress and the Self-Regulatory Model) [22,23]. A teachable moment event is thought to motivate individuals to adopt risk-reducing behaviors that will improve their health or reduce their risk of adverse health outcomes [18]. It is possible that HIV events/stages serve as “teachable moments” (McBride) when PLWHA have increased motivation and self-efficacy, leading to an increased likelihood of smoking cessation. An HIV-related event, such as the initiation of HIV care, will trigger a cognitive stress response and increase one’s perceived impact of HIV, leading to an increase in risk perception, motivation to quit smoking and beliefs that smoking cessation with improve HIV-related health. These same attitudes about smoking are expected to directly increase intentions to quit smoking, to facilitate cessation attempts and increase the likelihood of successful cessation.

Because we hypothesize that the HIV diagnosis (or care initiation) elicits the greatest perceived impact during an individual’s HIV disease course, we expect this event to be associated with the largest change in smoking outcomes (i.e., the highest number of quit attempts, stronger intentions to quit and higher rates of cessation). We further hypothesize that the time of disease progression - identified by treatment failure or diagnosis of an HIV-related disease - is associated with high levels of perceived impact (but not as high as the time of HIV diagnosis) and also elicits changes in smoking outcomes. Finally, when the disease is stable, the perceived impact of HIV is expected to be relatively low compared to the time of HIV diagnosis and HIV disease progression. Thus, it is unlikely for changes in smoking outcomes to be observed during periods of HIV disease stability.

The framework also suggests that individuals who find an HIV-related event (e.g., HIV diagnosis) to have little impact on their lives will be unlikely to experience a change in smoking attitudes or smoking outcomes. Finally, the framework suggests that two domains, subjective norms and perceived control, will moderate the relationship between the perceived impact of HIV and smoking outcomes. For example, an individual who perceives an event to be of high impact and has a high level of self-efficacy will be more likely to make a quit attempt than a participant who perceives a high impact of disease but has a lower level of self-efficacy.

### Measures

#### Perceived impact of HIV/AIDS

Two measures are used to assess the participant’s perceived impact of HIV/AIDS. *The Brief Illness Perception Questionnaire (Brief-IPQ)* is used to measure cognitive and emotional illness representations. This short 8-item measure was designed to evaluate key domains (i.e., consequences, personal control, treatment control, identity, concern, and understanding) involved in an individual’s response to a health threat, as described by Leventhal’s Self-Regulatory Model [23]. This scale has solid psychometric properties, and has been used with numerous disease populations, including PLWHA [24,25].

The 22–item *Impact of Events Scale-Revised (IES-R)* is used to assess the participant’s level of perceived distress (during the past 7 days) due to HIV. The IES-R can be scored to generate three subscales (Avoidance, Intrusions, and Hyperarousal) and an overall score. This scale has solid psychometric properties and has been used with many diverse populations [26].

#### Attitudes about smoking

Several measures are used to assess attitudes about smoking. Quit motivation is assessed with the *Reasons for Quitting* questionnaire. This 20-item scale assesses intrinsic (health concerns, self-control) and extrinsic (immediate reinforcement, social influence) motives for quitting smoking. Both intrinsic motives and the ratio of intrinsic to extrinsic motives have been demonstrated to predict successful smoking cessation [27,28]. To assess risk perceptions and outcome expectancies, we developed a series of items based on recommendations by Brewer and colleagues [29]. Specifically, participants respond to the following four questions, as applicable: 1) “If you don’t quit or go back to smoking, what are your chances of ever developing a smoking-related health problem?”; 2) “If you quit smoking or remain quit, what are your chances of ever developing a smoking-related health problem?”; 3) Compared to other smokers, what are your chances of ever developing a smoking-related health problem if you continue or go back to smoking?”; 4) Compared to other smokers, what are your chances of ever developing a smoking-related health problem if you quit smoking or remain quit?”

#### Perceived control

Three separate measures are used to assess perceived control. The widely used and well-validated 18-item *Multidimensional Health Locus of Control (MHLC)* scale is used to assess internal and external locus of control [30,31]. In addition, *Treatment Involvement* is measured by a 4-item scale used on the HIV Cost and Services Utilization Study [32]. These items measure desire for information involvement and preference for decision involvement in one’s own care. Item responses are scored on a 4-point scale ranging from strongly agree to strongly disagree [33]. Finally, the 9-item self-efficacy scale developed and validated by Velicer and colleagues is administered [34]. This commonly used self-efficacy scale assesses an individual’s confidence in his/her ability to not smoke in a variety of situations.

#### Subjective norms

We constructed three sequential questions to assess subjective norms. The questions were constructed based on the recommendations of Ajzen [35]. An example of these items is, “My family thinks that it is important for me to quit smoking”. Identically structured items will substitute “family” with “friends” and “doctor”. Each item will be scored on a 7-point Likert scale ranging from 1-extremely likely to 7-extremely unlikely.

#### Intention to quit smoking

Intention to quit smoking is assessed by asking participants whether or not they plan to quit smoking. Responses are entered on a 7-point scale, ranging from 1-definitely no to 7-definietely yes. An additional intention item considers time frame (i.e., quitting within the next month - within the next year). Similar items assess intention to cut back on the number of cigarettes smoked per day, and time frame for planned reduction.

In addition to these intention items, we assess the participant’s readiness to quit with two commonly used measures: *Stage of Change and the Contemplation Ladder. Stage of Change* is a widely used approach that categorizes individuals into stages (e.g., pre-contemplation, contemplation, action, and maintenance) based on past quit attempts and timing of planned future quit attempts [36]. *The Contemplation Ladder* is a single item that asks respondents to circle a number on a 10-rung ladder that represents their current level of readiness to consider smoking cessation [37]. Responses range from 0 (no thought of quitting) to 10 (taking action to quit smoking, e.g. cutting down enrolling in a cessation program).

#### Smoking status

*The Smoking Status Questionnaire* is a 10-item questionnaire that assesses smoking behavior within the last seven days, within the last 30 days, and since the time of last contact. Cigarettes smoked per day, longest period of abstinence since last contact, number of relapses, use of nicotine replacement, exposure to other types of tobacco, or use of any other cessation treatment (e.g., professional assistance and self-help) are also included [38].

#### Demographic, health, and smoking questionnaires

These items are designed to provide demographic data (e.g., age, race/ethnicity, education level, income, and occupation), current medications, current medical care (including number and type of healthcare visits), route of HIV infection, drug/alcohol use, history of depression, and smoking history (e.g., years smoked, amount smoked, age of initiation, previous quit attempts, and relapse history). These items have been used in several of our other smoking cessation trials as well as in our pilot study [39-41].

#### Fagerström test for nicotine dependence (FTND)

The original items of the *Fagerström Tolerance Questionnaire (FTQ)* were derived from theoretical conceptualizations of reliance on nicotine [42]. The instrument is reliable and useful in a broad spectrum of populations [43]. The *FTND*, a modification of the FTQ, is a 6-item scale with solid psychometric properties [44].

#### Center of epidemiologic studies depression scale (CES-D)

The *CES-D* is a 20-item measure developed to assess depressive symptoms in community, non-clinical populations [45]. This scale consists of four major factors: depressed affect, enervation, lack of positive affect and interpersonal problems. Good psychometric properties have been demonstrated across diverse populations [7].

#### Positive and negative affect schedule (PANAS)

The *PANAS* is a 20-item adjective rating form that includes both positive and negative affect scales. Ratings are based on a five-point Likert scale (1 = very slightly or not at all to 5 = extremely). The scales have demonstrated high reliabilities, and intercorrelations between the scales are low [46].

#### Interpersonal support evaluation list (ISEL)

The *ISEL* is used to measure social support. This 12-item measure assesses three constructs of social support: tangible, appraisal, and belonging. Social support is a well-established predictor of successful smoking cessation [47].

#### AIDS clinical trials group (ACTG) measures

Two measures developed by the ACTG are used to capture both HIV/AIDS-related symptom status and HIV medication adherence. The *HIV Symptom Index* is a 20-item instrument that assesses the presence of 20 HIV-related symptoms, and the degree to which these symptoms are distressing to the respondent. The scale has been validated and correlates with measures of functional status and HIV disease stage [48]. Using this measure in our previous work, we have shown that smoking cessation can significantly reduce symptom burden [49]. The 20-item *HIV Medication Adherence Questionnaire* assesses overall adherence to HIV medications, and variables associated with adherence (e.g., away from home, side effects, and complication of regimen) [50].

#### Mental status

We use the *Mini-Mental Status Examination (MMSE)* to assess possible cognitive impairment. This brief measure is widely used in a variety of clinical and research settings and has been validated in English and Spanish [51]. See Table 1 for assessment schedule.

**Table 1:** Assessment schedule

**Measure**	**Baseline**	**Cell Phone assessments weeks 1-12**	**Follow-up assessments**			
			**3-month**	**6-month**	**9-month**	**12-month**
Brief Illness perception questionnaire (Brief-IPQ)	X	X	X	X	X	X
Impact of events scale (IES-R)	X		X	X	X	X
Reasons for quitting	X	X*	X	X	X	X
Risk perceptions/qutcome expectancies	X	X	X	X	X	X
Health locus of control (MHLC)	X		X	X	X	X
Self-efficacy	X		X	X	X	X
Treatment involvement	X		X	X	X	X
Subjective norms	X		X	X	X	X
Intention to quit smoking	X	X*	X	X	X	X
Contemplation ladder	X		X	X	X	X
Stage of change	X		X	X	X	X
Smoking status (self-report)		X*	X	X	X	X
Demographics and health hehaviors	X		X*	X*	X*	X*
Fagerström test for nicotine dependence (FTND)	X		X	X	X	X
Center of epidemiologic studies depression scale (CES-D)	X		X	X	X	X
Positive and negative affect schedule (PANAS)	X		X	X	X	X
Interpersonal support evaluation list (ISEL)	X		X	X	X	X
ACTG symptom status	X		X	X	X	X
ACTG medication adherence			X	X	X	X
Expired carbon monoxide	X		X	X	X	X
Mini-mental status examination (MMSE)	X		X	X	X	X

*Brief version.

### General analytic approach

Because we are collecting repeated measurements that are correlated within subjects, our data analytic approach utilizes generalized linear mixed model (GLMM) regression [52,53]. GLMM is a flexible analytic approach with wide use in health sciences research [54]. It can handle fixed- and random-effect model parameters, as well as both nested designs and repeated measures with various correlation structures [52,53]. GLMM can also handle normal and non-normal outcomes (such as the dichotomous outcome of smoking cessation), different variance functions, as well as unbalanced designs where the number of repeated measurements varies across individuals.

### Statistical analysis

Aim 1 is to assess the relationship between HIV disease event/stage (i.e., diagnosis, stable, and progressive disease) and smoking outcomes (i.e., intention to quit, number of quit attempts, and cessation outcomes). To evaluate Specific Aim 1, we will assess each of the pairwise comparisons between the three disease events/stages. For the primary analysis, intention to quit smoking will be classified as a dichotomous endpoint. Specifically, participants who have either quit smoking or report high levels of intention to quit smoking (i.e., scores of 5–7 on the 7-point intention-to-quit scale) will be compared to participants who do not intend to quit (i.e., current smokers with scores of 1–4 on the intention to quit scale). A GLMM with binomial error distribution and logit link function will be used for this analysis. The same analysis will be used for the binary cessation outcome. In this model, we will use 7-day point prevalence abstinence as our outcome. That is, participants who self-report no smoking (not even a puff) in the past seven days and have an expired CO level of < 10 ppm will be considered abstinent. Similarly, the number of quit attempts (i.e., trying to quit and remaining abstinent for ≥ 24 hours) will be analyzed using a Poisson mixed effects model, a special class of GLMMs, and continuous cessation outcomes will be analyzed using a linear mixed model.

Aim 2 is to evaluate perceived impact of HIV as a potential mediator of the association between disease stage and smoking outcomes. We will assess the indirect effects of disease event/stage on smoking outcomes (binary, ordinal or continuous) through perceived impact of HIV, not necessarily requiring that a significant association between disease event/stage and smoking outcomes be found, as long as the hypothesized theory in our conceptual framework calls for the analysis. This strategy is consistent with new recommendations regarding mediation analysis in social psychology [55]. Exploratory analyses will also be performed to assess the mediation and/or moderation effects of all the constructs in the conceptual framework. To assess the mediation effects, we will use the approaches of MacKinnon and colleagues and Preacher and Hayes [56-60], as appropriate. In particular, we will use a bootstrap resampling approach to calculate the confidence intervals of the indirect effects. Moreover, for binary and ordinal outcomes, we will follow the approaches recommended by MacKinnon and colleagues [57,61].

Aim 3 is to evaluate potential mediators (i.e., attitudes about smoking) and moderators (i.e., perceived control and social norms) of the relationship between perceived impact of HIV disease and smoking outcomes. We will assess the indirect effects of perceived impact of HIV on smoking outcomes through attitudes about smoking. Similar procedures for testing for mediation effects for Specific Aim 2 will be employed in assessing Specific Aim 3. In addition, we will assess whether perceived control and social norms moderate the above association, by including and testing the interaction between these variables and the perceived impact of HIV in the GLMMs. A significant interaction effect will indicate the presence of a moderation effect.

Aim 4 is to describe, in detail, the smoking behavior of individuals recently diagnosed with HIV within the first three months after study enrollment. For Specific Aim 4, we expect that the probability of intention to quit smoking will decrease over the three-month period after initial diagnosis. A series of GLMMs will be generated. We will first use GLMM to model parametric time profiles of this smoking behavior. In the event that the data suggest a nonlinear trajectory, nonparametric or nonlinear regression models for longitudinal data will be explored or employed. In particular, models that may be useful include, for example, Zhang et. al. [62], Lin and Zhang [63], and Li et al. [64]. These methods are applicable to normal (continuous) or non-normal (e.g., categorical) smoking outcome data. Estimation of such time profiles will allow us to determine the teachable moment’s magnitude and duration in this critical period of time following HIV diagnosis. In the second step, separate models will be generated to explore the effects of perceived impact of HIV, risk perception, and quit motivation, in addition to time, on quit intention. A final step will include all three predictor variables in a single model. It should also be noted that potential confounders (e.g., smoking cessation treatment) will be controlled in each step. We will perform a similar series of analyses to consider current smoking status.

#### Missing data and drop-outs

Some participants will fail to complete all planned assessments and consequently present missing data. For missing smoking outcomes, we will use a conservative approach of treating missing intention-to-quit or cessation outcome data as no intention to quit smoking, or imputing the number of quit attempts with the currently observed number (i.e., no improvement after that). While this generally is considered a conservative approach, we note that GLMM will give unbiased estimates of effects provided that the probability of having missing data depends only on the observed variables in the model (or are missing at random [MAR]). We will conduct additional sensitivity analyses assuming different missing data mechanisms. For example, we will consider a multiple imputation approach based on relevant patient characteristics at baseline to account for potential missing at random (MAR) mechanisms. We will also explore pattern-mixture and selection models to account for potential missing not at random (MNAR) mechanisms [65]. Similar findings based on these analyses will strengthen our study conclusions.

#### Power and sample size

We present a power analysis for Primary Aim 1 based on several conservative estimations. Specifically, the following assumptions are made: 1) Approximately 50% of patients will intend to quit smoking at the time of initial HIV diagnosis. This is based on earlier reports that >50% of HIV patients report adopting healthier behaviors following an HIV diagnosis [33]. At each follow-up visit, patients will be classified as having stable HIV disease or progressive HIV disease based upon their viral load, CD4 count and presence of AIDS-defining illnesses; 2) It is anticipated that 30% of participants will meet the criteria for progressive disease in the 12-month follow-up window.

We will enroll 450 participants in the study, and conservatively estimate that 20% of these participants will miss at least one of the four (i.e., 3-, 6-, 9-, and 12-month) follow-up measurements. Assuming that 50% of participants will quit or intend to quit at diagnosis, using a logistic mixed model analysis we will have 87% power to detect a rate to quit or intent to quit of 39% or lower in progressive disease and 99% power to detect a rate of 30% in patients with stable disease. We will have 88% power to detect the difference between 30% and 39% in the stable and progressive arms. Conservatively, we have chosen to base our power estimates on a dichotomous outcome, as described above. Power will generally be higher when the outcome variable is ordinal or continuous. Therefore, we will have excellent power to detect moderate effect sizes for Primary Aim 1.

## Discussion

Efforts to develop and assess novel smoking cessation treatments for underserved HIV-positive populations are needed. By evaluating events/stages of HIV disease as potential teaching moments for smoking cessation, findings from this study could be used to develop treatments tailored to an individual’s stage of HIV disease. We expect that disease events/stages will influence perceived impact of HIV disease which will, in turn, influence smoking related attitudes and behavior. For example, if individuals are more likely to be motivated to make a quit attempt at the time of HIV diagnosis, efforts to target individuals at this time with relatively low intensity treatments may be quite effective. On the other hand, treatments targeting individuals with stable HIV disease - who may have relatively low levels of motivation to quit - may require greater intensity.

**Figure 2:**
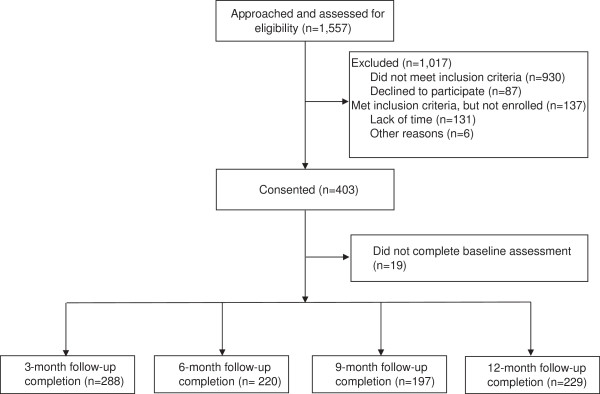
CONSORT diagram of Project STATE: screening, study enrollment, and retention through 12-month follow-up.

Thus far, we have successfully consented 403 participants. See Figure 2 for the CONSORT (Consolidated Standards of Reporting Trials) diagram which displays participant recruitment, enrollment and follow-up to date. Study participants are predominantly male (72.9%), Black/African-American (63.8%), have completed 10.9 years of formal education, were infected through heterosexual contact (45.6%), are smoking 10 or fewer cigarettes per day (60.2%) and have a mean age of 38.7 years. See Table 2 for complete demographic and psychosocial and smoking variables of study participants.

**Table 2:** Baseline characteristics of study participants

**Demographic, psychosocial and smoking variables**	**n = 384**	
Mean age in years (SD)	38.7 (10.6)	
Male, n (%)	280 (72.9)	
Married/living with significant other, n (%)	70 (18.2)	
Race/ethnicity, n (%)		
White	69 (18.0)	
Black/African American	245 (63.8)	
Latino/Hispanic	56 (14.6)	
Other	14 (3.6)	
Mean years of formal education (SD)	10.9 (4.0)	
Education level, n (%)		
Less than high school	139 (36.2)	
High school or equivalent	150 (39.1)	
More than high school	95 (24.7)	
Current employment status, n (%)		
Working full or part time	63 (16.4)	
Not working due to health	185 (48.2)	
Unable to find work	95 (24.7)	
Not working for other reasons	41 (10.7)	
HIV transmission, n (%)		
Male homosexual contact	132 (34.4)	
Heterosexual contact	(45.6)	
Injection drug use	35 (9.1)	
Other	42 (10.9)	
Depression (CES-D score^1^), mean (SD)	22.1 (11.8)	
Cigarettes smoked per day, n (%)		
10 or fewer	231 (60.2)	
11 to 20 per day	117 (30.5)	
21 to 30 per day	19 (4.9)	
31 or more per day	17 (4.4)	
Nicotine dependence (FTND score^2^), mean (SD)	4.3 (2.5)	
Alcohol use (AUDIT score^3^), mean (SD)	8.6 (7.6)	
Illicit drug use in past 30 days, n (%)	178 (46.4)	

^1^Range 0-60, scores ≥16 indicate risk of clinical depression.

^2^Range, 0–10.

^3^Scores ≥8 are associated with harmful or hazardous drinking.

This prospective study design enables us to carefully track changes in smoking behavior over time, and to link these changes to both the course of HIV disease and/or to the participant’s perceived impact of HIV. By identifying optimal time points for intervention, the findings from this study will have the potential to maximize the efficiency and efficacy of cessation treatments delivered in resource-limited settings. In addition, the findings will be instrumental in identifying specific constructs that should be targeted for intervention and will provide a strong foundation for the development of future cessation interventions targeting smokers living with HIV/AIDS.

## Abbreviations

PLWHA: People living with HIV/AIDS; HAART: High active antiretroviral therapy; HPV: Human papilloma virus; DAD: Data collection on adverse events of Anti-Hiv Drugs; STATE: Study of tobacco attitudes and teachable events; TSHC: Thomas street health center; ACASI: Audio computer assisted self-interview; CO: Carbon monoxide; Brief-IPQ: Brief illness perception questionnaire; IES-R: Impact of events scale-revised; MHLC: Multidimensional health locus of control; SSQ: Smoking status questionnaire; FTND: Fagerström test for nicotine dependence; CES-D: Center of epidemiologic studies depression scale; PANAS: Positive and negative affect schedule; ISEL: Interpersonal support evaluation list; ACTG: AIDS clinical trials group; MMSE: Mini-mental status examination; GLMM: Generalized linear mixed model; MAR: Missing at random; MNAR: Missing not at random; CONSORT: Consolidated standards of reporting trials.

## Competing interests

The authors declare that they have no competing interests.

## Authors’ contributions

DV and EG conceptualized and designed the study and were involved in drafting the manuscript. FF and MB prepared the manuscript draft and both contribute to study implementation. YL and RA contribute to study design and drafting the manuscript. All authors read and approved the final manuscript.

## Pre-publication history

The pre-publication history for this paper can be accessed here:

http://www.biomedcentral.com/1471-2458/14/149/prepub
